# WISP1 Inhibits Hepatocellular Carcinoma Cell Proliferation by Promoting CyclinD1 Ubiquitination and Downregulating its Expression

**DOI:** 10.5152/tjg.2024.23524

**Published:** 2024-12-16

**Authors:** Jinlong Yan, Jun wen Hu, Na Cheng, Nuoya Li, Anqi Xin, Zhipeng Wu, Zhengyi Wu, Jun Lei, Shouhua Zhang, Jinping Yao

**Affiliations:** 1 Department of General Surgery, The Second AffiliatedHospital, Jiangxi Medical College, Nanchang University, Jiangxi, China; 2 Department of Hospital Infection Control, The First Affiliated Hospital of Nanchang University, Jiangxi, China; 3 Department of Hepatobiliary Surgery, The Third Affiliated Hospital of Sun Yat-sen University, Guangzhou China; 4 Department of Operation Room, The Second Affiliated Hospital, Nanchang University Jiangxi Medical College, Jiangxi, China; 5 Department of Endocrinology, the Fourth Affiliated Hospital of Nanchang University, Jiangxi, China

**Keywords:** WISP1,, hepatocellular carcinoma, CyclinD1, Ubiquitination

## Abstract

**Background/Aims:**

Hepatocellular carcinoma (HCC), a leading cause of cancer-related deaths, is often linked to dysregulated cell cycle proteins. This study focuses on the role of WISP1 in modulating Cyclin D1, a key cell cycle regulator, in HCC.

**Materials and Methods:**

The study used HCCLM3 and Hep3B cells to assess the expression of Cyclin D1 and cell proliferation following the treatment of WISP1. This was achieved through Western blot, qRT-PCR, and EdU assays. Additionally, animal studies were conducted to evaluate the effects of WISP1 treatment on Cyclin D1 expression and cell proliferation.

**Results:**

Overexpression of WISP1 in HCC cells led to a marked decrease in Cyclin D1 protein levels and reduced cell proliferation. WISP1 influences Cyclin D1 through post-translational modifications, particularly ubiquitination and proteasomal degradation.

**Conclusion:**

The findings revealed that WISP1’s modulation of Cyclin D1 plays a critical role in inhibiting HCC cell growth, highlighting a potential therapeutic target for HCC treatment.

**Main Points**

- WISP1 overexpression inhibits HCC cell proliferation both in vivo and in vitro.
- WISP1 suppresses HCC cell proliferation by reducing Cyclin D1 expression.
- In HCC, the degradation of Cyclin D1 relies on the ubiquitin-proteasome system.
- Overexpressing WISP1 in hepatoma cells accelerates the ubiquitination and degradation of the Cyclin D1 protein.

## Introduction

Hepatocellular carcinoma (HCC), a prevalent malignant tumor, ranks as the sixth most common cancer and the third leading cause of cancer-related deaths worldwide.^1^ The late diagnosis of HCC often limits the effectiveness of treatment options, resulting in only about 20% of patients being eligible for radical resection.^2^ The progression of HCC is intricately linked to the abnormal expression of cancer-related genes.^3^

A critical protein in the pathology of HCC is WISP1 (Wnt-induced secreted protein 1), which is involved in the Wnt signaling pathway. WISP1 plays a vital role in cellular processes such as proliferation and apoptosis.^4,5^ A previous study has revealed that WISP1 expression varies among different cancer types. It is found to be overexpressed in certain cancers, including oral squamous cell carcinoma and colorectal cancer, contributing to tumor growth.^6,7^ In contrast, WISP1 is under-expressed in other types of cancers, such as melanoma, lung, and breast cancer, leading to different growth behaviors in these tumors.^9-11^ In HCC, our research has indicated a reduction in WISP1 expression, implying its role in suppressing tumor proliferation.^12^

Cyclin-D1, encoded by the CCND1 gene, is another significant protein involved in the regulation of the cell cycle and is linked to the progression of various cancers.^13^ The expression of Cyclin D1 is influenced by oncogenes in gastric cancer.^15^ and by microRNAs in glioblastoma.^16^ Studies have shown that suppressing Cyclin D1 in HCC can lead to a reduction in cell growth,^17^ and therapies such as sorafenib target this protein to combat tumor proliferation.^18^ Evidence suggests that WISP1 has an effect on the levels of Cyclin D1 in HCC,^12^ but the specific nature of this interaction is not yet fully understood.

This study aims to delve into the relationship between WISP1 and Cyclin D1 in HCC, especially focusing on how the modulation of Cyclin D1 by WISP1 impacts the growth of HCC cells. This investigation seeks to provide insights into potential therapeutic targets for HCC.

## Materials and Methods

### Cell Culture

Hep3B and HCCLM3 HCC cells were obtained from the Shanghai Cell Bank of the Chinese Academy of Sciences. The cells were grown in DMEM complete medium supplemented with 10% fetal bovine serum and dual antibiotics (100 units/mL penicillin and 100 μg/mL streptomycin, sourced from Solarbio, China). The cultures were maintained at 37°C in a humidified atmosphere with 5% CO_2_. The media was refreshed every 2 days followed by sub-culturing at a 1: 3 ratio. Experiments were conducted when cells reached approximately 80% confluency.

### qRT-PCR

Using TRIzol reagent from Boster, China, total RNA was isolated from the cells following the provided guidelines. The primers employed in this research were: Cyclin D1 - Forward: 5-gctgtctctttgtctctgccc-3, Reverse: 5-tggaaggtgttctcaagggg-3; GAPDH - Forward: 5-ggagcgagatccctccaaaat-3, Reverse: 5-ggctgttgtcatacttctcatgg-3.qRT-PCR was performed as we reported previously.^12^

### Western Blot Analysis

Cells were harvested and lysed in RIPA buffer (Biyuntian, Shanghai, China) supplemented with protease inhibitors for protein extraction. Western blotting was performed as we reported previously.^12^

### Plasmids and Reagents

Based on the sequences of WISP1 (NM_080838) and Cyclin D1 (NM_053056),the WISP1 overexpression plasmid GV341-WISP1-His and Cyclin D1 overexpression plasmid GV341-Cyclin D1-Flag (Jikai Company, Shanghai) were designed and constructed. Hep3B and HCCLM3 cells were transfected with plasmids that overexpress and downregulate the target gene. Hep3B and LM3 cells transfected with empty plasmid Vector-His or Vector-Flag were used as controls.

### Cells Proliferation Analysis: EdU and MTT

Cell viability was determined using a 5-ethynyl-20-deoxyuridine (EdU) assay, following the protocol recommended by the manufacturer (RiboBio) as documented in previously published studies.^19^ The MTT Cell Proliferation/Viability Assay Kit from R&D systems was used to evaluate cell survival, with procedures strictly adhering to the manufacturer’s guidelines and as detailed in prior research.^20^

### Co-Immunoprecipitation

Treated cells were lysed using RIPA buffer with PMSF protease inhibitor from Boster, China. The specific experimental steps were performed as we reported previously.^12^ Finally, immune complexes were separated by SDS-PAGE and detected by WB analysis.

### Protein Degradation and Half-Life Determination

For protein degradation measurements, Hep3B cells and LM3 cells were exposed to 15 µM of the proteasome inhibitor MG132 (Sigma, St. Louis, USA) for the indicated times. For half-life measurements, Hep3B cells and LM3 cells were exposed to 15 µM cycloheximide (Sigma). Cycloheximide is a protein biosynthesis inhibitor that acts for a specified time. Proteins were collected and analyzed by WB.

### In Vivo Experiments

BALB/C nude mice, aged 4-6 weeks and weighing 18-20 g (n = 14), were acquired and maintained in a sanitized setting with regulated lighting and temperature conditions. HCCLM3 cells were transfected with GV341-vector and GV341-WISP1-his, respectively. The cells of each group in the logarithmic growth phase were taken, and the cell concentration was adjusted to 107/mL. About 200 μL cancer cell suspension was inoculated on the back of each nude mouse with a 1 mL syringe. Starting from day 3 post-inoculation, the dimensions of the subcutaneous tumor, including length, width, and thickness, were determined every 3 days using a vernier caliper. The tumor volume was estimated using the formula (L × W^2^)/2. On the 45th day, all mice were anesthetized and subsequently euthanized. The tumors from the mice’s back were then removed and weighed. Results are expressed as mean ± SD (mean ± SD). The animal study received approval from the Animal Ethics Committee of the Second Affiliated Hospital of Nanchang University (approval no. 2021011; date: December 1, 2022).

### Statistical Analysis

All data were analyzed using GraphPad Prism v7 software (GraphPad Software, Inc., Boston, USA). Differences between groups were analyzed by the Student’s *t* test. More than 2 groups were compared, and one-way analysis of variance (ANOVA) was used to compare between groups. *P* < .05 or .01 was considered significant. All experiments were replicated independently at least 3 times, and data are presented as mean ± SD.

## Results

### Overexpression of WISP1 Inhibits HCC Cell Proliferation Both In Vitro and In Vivo

**Figure 1.**
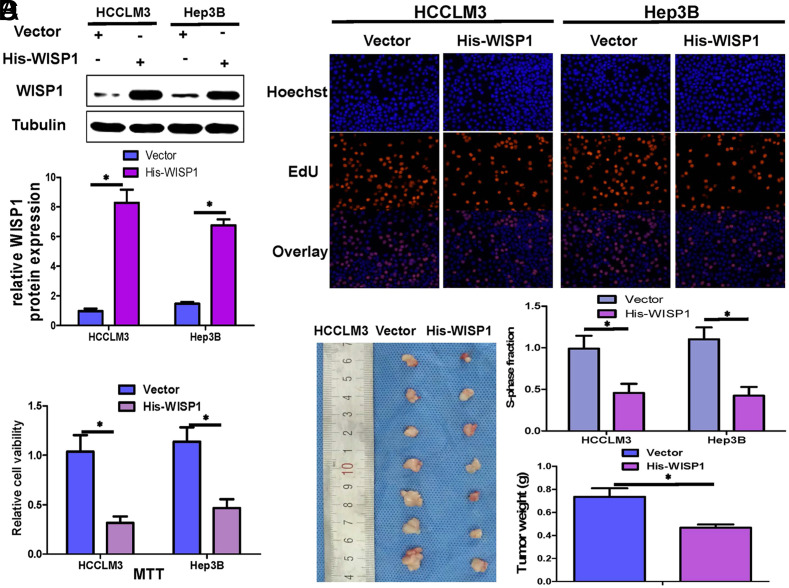
Overexpression of WISP1 inhibits HCC cell proliferation in vitro and in vivo.

To elucidate the impact of WISP1 overexpression on hepatoma cell growth, we introduced WISP1 overexpression plasmids into HCCLM3 and Hep3B cells. The overexpression of WISP1 was confirmed through Western blot analysis (Figure 1A).The growth rate of hepatoma cells was assessed using MTT and EdU tests. Methyl Thiazolyl Tetrazolium assay outcomes suggested a notable decline in HCC cell activity in the WISP1 overexpression group (Figure 1B). Similarly, the EdU test revealed that the proportion of EdU-positive HCC cells in the WISP1 overexpression group was significantly lower compared to the control (Figure 1C). At the same time, we further constructed a subcutaneous transplanted tumor model of HCCLM3 and compared the tumor weight of the control group and the WISP1-overexpressing group. The findings revealed that tumors with WISP1 overexpression had notably reduced weights compared to the control group (Figure 1D). These observations suggest that WISP1 overexpression hampers the growth of hepatoma cells both in laboratory conditions and in living organisms.

### WISP1 Inhibits HCC Cell Proliferation by Downregulating Cyclin D1 Expression

**Figure 2.**
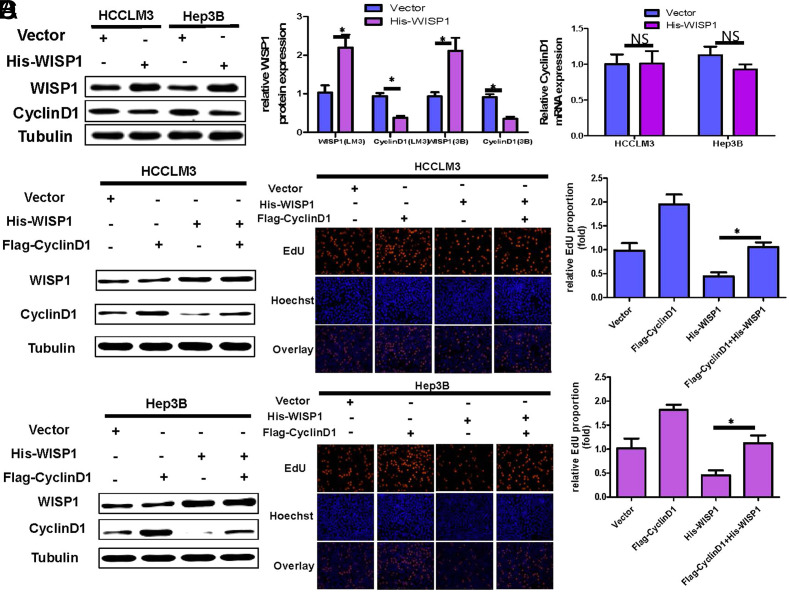
WISP1 inhibits HCC cell proliferation by downregulating Cyclin D1 expression.

To determine if WISP1 suppresses hepatoma cell proliferation through the modulation of cyclin D1, we introduced an overexpression of WISP1 in these cells using the WISP1-his plasmid. The expression levels of Cyclin D1 protein and mRNA were assessed using WB and qRT-PCR, respectively. The results showed that Cyclin D1 protein was significantly down-regulated (Figure 2A), but Cyclin D1 mRNA expression was not significantly changed (Figure 2B). Therefore, WISP1 may regulate the expression of Cyclin D1 protein through post-translational modification. Given Cyclin D1’s role in controlling HCC cell growth, we hypothesize that Cyclin D1 might act as a mediator for WISP1’s inhibitory effect on HCC cell proliferation. To test our hypothesis, we conducted a recovery experiment. In HCCLM3 and Hep3B cells that overexpressed WISP1, we observed a significant decrease in Cyclin D1 protein expression. Furthermore, the results from the EdU assay indicated a notable reduction in the proliferation rate of hepatoma cells.(Figure 2C and D). However, overexpression of Cyclin D1 at the same time as overexpression of WISP1 restored the proliferative activity of hepatoma cells (Figure 2C and D). The results suggest that WISP1 curbed HCC proliferation by downregulating Cyclin D1 protein expression.

### Degradation of Cyclin D1 in HCC is Dependent on Ub Proteasome

**Figure 3.**
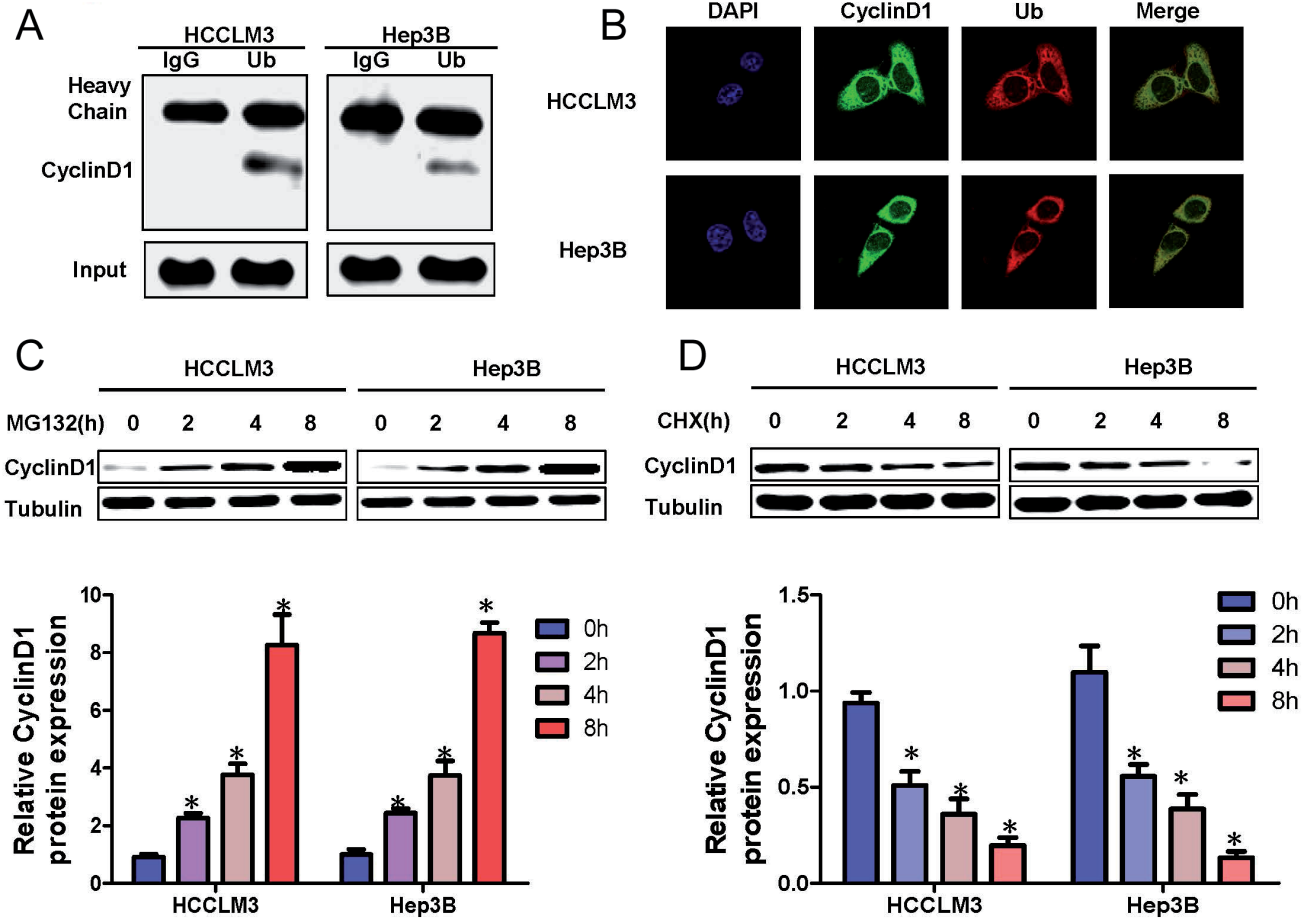
Degradation of Cyclin D1 in HCC is dependent on Ub proteasome.

Ubiquitin-proteasomal degradation is the main pathway for protein post-translational modification regulation.^22^ It remains unknown whether Cyclin D1 protein can be modified by polyubiquitination and rapidly degraded by the ubiquitin-proteasome system. To confirm that Cyclin D1 protein level regulates the ubiquitination-proteasomal degradation in hepatoma cells, we first confirmed whether Cyclin D1 interacts with Ub in Hep3B and HCCLM3 cells. Co-immunoprecipitation experiments revealed that Cyclin D1 associates with Ub (Figure 3A), and laser confocal studies confirmed their co-localization (Figure 3B). Thus, Cyclin D1 in Hep3B and HCCLM3 cells can bind to Ub; MG132 is a specific blocker of the ubiquitin-proteasome degradation pathway. In order to verify whether Cyclin D1 is degraded by ubiquitin-proteasome, the proteasome inhibitor MG132 was added to the Hep3B and HCCLM3 cells. By Western blot detection, it was found that the expression of Cyclin D1 gradually increased with time (Figure 3C). Additionally, cycloheximide (CHX) can inhibit protein synthesis by blocking mRNA translation.^23^

Upon exposure to cycloheximide (CHX), Cyclin D1 expression in Hep3B and HCCLM3 cells diminished progressively over time (Figure 3D).The above results indicate that Cyclin D1 is degraded by proteasome Ub in hepatoma cells.

### Overexpression of WISP1 in Hepatoma Cells Promotes Ubiquitination and Degradation of Cyclin D1 Protein

**Figure 4.**
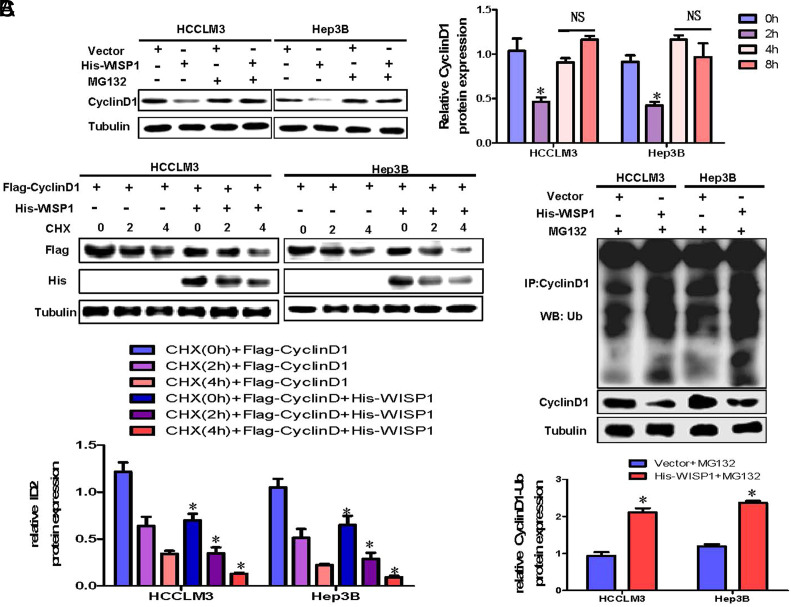
Overexpression of WISP1 in hepatoma cells promotes ubiquitination and degradation of Cyclin D1 protein.

Prior research has revealed that WISP1 suppresses the growth of HCC cells by reducing Cyclin D1 protein levels, and Cyclin D1 is subject to degradation via the Ub-proteasome pathway. Therefore, we speculate that WISP1 may inhibit its protein expression level by promoting Cyclin D1 ubiquitination and degradation. Elevating WISP1 levels in HCC cells led to a decline in Cyclin D1 protein expression. However, upon adding the proteasome inhibitor MG132 along with WISP1 overexpression, the Cyclin D1 protein levels remained relatively stable (Figure 4A). To examine the effect of overexpressing WISP1 on the degradation rate of Cyclin D1 protein, HCCLM3 and Hep3B cells were co-transfected with His-WISP1 and Flag-Cyclin D1, respectively. The degradation level of Cyclin D1 was detected by WB with and without CHX, respectively. Findings indicate that increased WISP1 expression facilitated the breakdown of Cyclin D1 (Figure 4B). To further verify whether overexpression of WISP1 promoted the ubiquitination level of Cyclin D1, we evaluated the changes in the ubiquitination level of Cyclin D1 by overexpression of WISP1 in the case of adding MG132 by WB. The data revealed that amplifying WISP1 expression notably elevated the ubiquitination of Cyclin D1 (Figure 4C). This suggests that heightened WISP1 expression enhances the ubiquitination and subsequent breakdown of Cyclin D1, leading to its reduced protein levels.

In conclusion, our study demonstrates that the overexpression of WISP1 plays a pivotal role in the suppression of hepatocellular carcinoma (HCC) cell proliferation. This inhibition occurs both in vivo and in vitro and is primarily mediated through the reduction of Cyclin D1 expression. Furthermore, we found that in HCC, the degradation of Cyclin D1 depends on the ubiquitin-proteasome system. Significantly, the overexpression of WISP1 in hepatoma cells leads to enhanced ubiquitination and subsequent degradation of the Cyclin D1 protein, further elucidating the molecular mechanisms underlying HCC cell proliferation inhibition.

## Discussion

Uncontrolled cell growth is a hallmark of HCC progression. WISP1 plays a critical role in maintaining normal cellular functions, including cell growth, adhesion, migration, and formation of the extracellular matrix.^5^ Although the implications of WISP1 in tumorigenesis remain debated, prior research indicates its notably reduced expression in HCC.^12^ Our current study reaffirms that increasing WISP1 expression significantly reduces hepatoma cell proliferation, as observed both in laboratory settings and in living organisms. Hence, WISP1 acts as an anti-cancer agent in liver malignancies.

Cyclin D1, pivotal for the G0/G1 phase transition in the cell cycle, manifests abnormal expression in numerous cancer cells and stands as an indicator of cancer characteristics and disease advancement.^23^ As highlighted by Hamada T et al., the primary recognized function of Cyclin D1 is to stimulate CDK4 and CDK6, which in turn enhances cell growth.^24^ Multiple studies have underscored the significant role Cyclin D1 holds in tumor proliferation.^14,15^ The oncogene HOXA1 amplifies gastric cancer cell growth by boosting Cyclin D1 expression;^15^ Li X and colleagues discovered that miR-340 curtails glioblastoma proliferation by repressing Cyclin D1 expression.^16^ Furthermore, BCL-3 enhances HCC growth by modulating cell proliferation and the cell cycle via cyclin D1.^17^ Sorafenib also suppresses HCC cell growth by downregulating Cyclin D1 expression.^18^ Thus, Cyclin D1 stands as a central pivot in controlling hepatoma cell proliferation. In our research, we delved deeper to confirm that WISP1 acts as an inverse regulator of Cyclin D1 protein levels. Moreover, through a rescue experiment, we established that Cyclin D1 is pivotal in mediating WISP1’s anti-proliferative impact on HCC cells.

Post-translational modification of proteins plays a crucial role in modulating their expression levels. Ubiquitination modification is a common protein post-translational modification. Typically, proteins that undergo ubiquitination are targeted for degradation via the ubiquitin-proteasome system, leading to a reduction in their expression levels. There is no research on the degradation pathway of Cyclin D1. Nathalie et al suggest that WISP1 may promote PPARγ proteasomal degradation by binding PPARγ, which in turn downregulates PPAR γ protein levels.^25^ Interestingly, this study found that WISP1 downregulated Cyclin D1 protein expression levels but did not affect WISP1 mRNA levels. Consequently, downregulation of Cyclin D1 protein expression by WISP1 may be related to Cyclin D1 ubiquitin-proteasome pathway degradation. This study confirmed that the degradation of Cyclin D1 in HCC cells is dependent on the Ub-proteasome pathway. Further experiments showed that overexpression of WISP1 significantly increased the ubiquitination level of Cyclin D1, thereby promoting the ubiquitination and degradation of Cyclin D1. In this study, we found no evidence of WISP1 binding to Cyclin D1 (data not publicly available). Consequently, the specific molecular mechanisms through which WISP1 promotes the ubiquitination of Cyclin D1 require further investigation. In conclusion, our findings revealed that WISP1 suppresses hepatoma cell proliferation through the downregulation of Cyclin D1 protein levels. Mechanistically, it was further found that WISP1 downregulated its protein expression by promoting the ubiquitination and degradation of Cyclin D1.

Based on previous studies, the molecular mechanism of WISP1 down-regulating Cyclin D1 protein level was further clarified, which is a continuation and in-depth extension of the previous studies. It provides theoretical support for WISP1 protein to inhibit the progression of liver cancer. However, there are some deficiencies in this paper: How WISP1 promotes the ubiquitination of cyclin D1 is not addressed, and it still needs further study. Therefore, the depth of research needs to be improved.

## Data Availability

The data that support the findings of this study are available on request from the corresponding author.
