# Erratum

**DOI:** 10.5152/tjg.2026.00261

**Published:** 2026-08-01

In the original article authored by Jinlong Yan, Jun Wen Hu, Na Cheng, Nuoya Li, Anqi Xin, Zhipeng Wu, Zhengyi Wu, Jun Lei, Shouhua Zhang, Jinping Yao, titled “WISP1 Inhibits Hepatocellular Carcinoma Cell Proliferation by Promoting CyclinD1 Ubiquitination and Downregulating its Expression” (Turk J Gastroenterol. 2025;36(4), doi: 10.5152/tjg.2024.23524.) published in the fourth issue of Turkish Journal Gastroenterology, the affiliation marker of author Jianping Yao was assigned incorrectly due to an oversight. The affiliation marker was published as 2, whereas the correct affiliation marker is 5. Subsequently, the affiliation marker was corrected, and the online version of the original article has been updated accordingly. You can access the revised version of this original article via the following link: https://turkjgastroenterol.org/index.php/tjg/article/view/4222

